# Inflammation and Anti-Inflammatory Targets after Aneurysmal Subarachnoid Hemorrhage

**DOI:** 10.3390/ijms22147355

**Published:** 2021-07-08

**Authors:** Sajjad Muhammad, Daniel Hänggi

**Affiliations:** Department of Neurosurgery, Heinrich-Heine University Medical Center, 40225 Düsseldorf, Germany; daniel.haenggi@med.uni-duesseldorf.de

**Keywords:** subarachnoid hemorrhage, inflammation, cytokines, post-SAH complications

## 1. Aneurysmal SAH and Sterile Inflammation after Aneurysm Rupture

Aneurysmal subarachnoid hemorrhage (aSAH), with a crude worldwide incidence of around 7.9 per 100,000 person-years [1] and accounting for only 5% of all strokes, is a potentially devastating cerebrovascular disease. Moreover, the mortality rate is extremely high and estimated to be approximately 50% (range 32% to 67%). Of particular concern is the fact that aSAH-affected patients are of much younger age than ischemic stroke [2,3,4], which results in a higher socioeconomic burden at the community level. Despite advancements in medical care, technical advances in aneurysm securement, and a decline in worldwide crude incidence of aneurysmal [1], the mortality and morbidity have not changed much over the last few decades. One-third of patients die within the initial week after the insult, and two-thirds of the survivors develop significant neurological or cognitive disabilities [3]. It is not yet completely understood which mechanisms contribute to this high mortality and morbidity. A lack of complete mechanistic understanding is the main reason why many pharmacological interventions fail to have a clinically meaningful effect. Hence, there is still a need to intensively explore and revisit the pathophysiology and underlying mechanisms at the cellular and molecular level.

Aneurysmal SAH is a multifactorial disease and multiple mechanisms and factors contribute to clinical outcome after aSAH. Delayed cerebral ischemia (DCI) is an important factor affecting about 30% of the survivors and seems to be a major contributor to the poor outcome after aSAH [3]. Until recently, cerebral vasospasm (CVS) was believed to cause DCI, but the latest research supports the notion that DCI is multifactorial and can be a consequence of multiple mechanisms, including altered autoregulation, cortical spreading depression (CSD), microthrombosis, and cerebral vasospasm (CVS) [5]. Systemic and local inflammation in the brain is a fundamental part of almost all processes contributing to DCI. Moreover, multiple central nervous system post-aSAH complications (such as cerebral vasospasm (CVS); hydrocephalus; seizures, meningitis; cortical spreading depression; and systemic complications, including peripheral infections, cardiomyopathy, and pulmonary edema) significantly contribute to the clinical outcome [6,7]. Here, both cellular inflammation and molecular inflammation play key roles in mediating all of these complications [8,9,10,11,12,13,14]. Hence, inflammation and inflammation-mediated processes might have great therapeutic potential to reduce the burden of DCI and other post-SAH complications and have an impact on the clinical outcome.

## 2. Initiators and Drivers of Inflammation after aSAH

In-depth knowledge of events, timing, and molecular/cellular mechanisms that initiate or sustain inflammatory processes are needed to be explored to identify suitable pharmacological targets. Rupture of an intracranial aneurysm causes blood to pour in subarachnoid space and leads to a transient increase in global intracranial pressure (ICP). This transient elevated ICP may lead to the release of molecules from damaged brain tissue [11,15]. Molecules from extravasated blood and from damaged brain, being the earliest events in the pathophysiology, seem to be the key initiators of the inflammatory cascade, including the expression of adhesion molecules and infiltration of immune cells (specifically macrophages) [11,13,15]. Infiltrated leukocytes and activated resident microglia start the inflammatory cascade, leading to the release of different inflammation-related cytokines [16]. This vicious cycle of inflammation probably contributes to almost all mechanisms in the course of aSAH, including apoptotic or necrotic cell death, cortical spreading depression (CSD), blood–brain barrier (BBB) disruption, microthrombosis, cerebral vasospasm (CVS), delayed cerebral ischemia (DCI), hydrocephalus, epilepsy, and multiple organ infections [3,6,7,8,9,10,11,12,13,14,15,16,17,18].

Due to the complex nature of the disease, it is difficult to mechanistically understand how exactly the inflammatory cascade initiates. Blood products and damage-associated molecular pattern molecules (DAMPs) released from damaged or stressed peripheral and central nervous system cells after the initial insult during the phase of early brain injury (EBI) [17,18,19] can potentially initiate the inflammatory cascade and connect to the delayed phase of post-aSAH complications [8,9,10,11,12,13,14,15]. Receptors of DAMPS are widely expressed in central nervous system cells, including endothelial cells, neurons, microglia, astrocytes, and infiltrating immune cells. The interaction of DAMPs with receptors, such as the receptor for advanced glycation end-products (RAGE), TLR-2, and TLR-4, may initiate and drive the inflammatory response in both the brain parenchyma and cerebral vessels as well as in systemic circulation; hence, this links EBI with delayed inflammation. EBI could be, in our opinion, an important and critical interval on the one hand contributing to acute mortality and on the other hand presenting an association with the post-EBI phase in survivors of aSAH.

## 3. Possible Novel Immune Pharmacological Approaches in aSAH

A decade and a half ago, the treatment and management of aSAH patients was solely focused on reversing cerebral vasospasm, which was considered a major factor leading to DCI and, hence, poor functional outcomes [20]. Therefore, numerous pharmacological approaches were developed to ameliorate CVS and consequent DCI, leading to improved outcomes. Consistent with this notion, several agents, such as statins, calcium channel blockers, nitric oxide donors, fasudil, cilostazol, magnesium sulphate, phosphodiesterase inhibitors, and endothelin receptor antagonists, were investigated to relieve the vasospasm [20,21]. Although these agents provided protection against CVS, most of them were found deficient in exerting beneficial effects regarding the clinical outcomes of patients [20,21]. Failure of clazosentan therapy to improve clinical outcomes led to the exploration of additional contributors to DCI and clinical outcome [20]. Only nimodipine and endovascular treatment of the ruptured intracranial aneurysms have been known to improve clinical outcomes [22]. Inflammation during EBI impacts the delayed events and clinical outcome [16,23,24]. Considering post-aSAH inflammation as a potential target, several approaches have great potential to improve the post-aSAH clinical outcome. DAMPs are initiators of the immune response; therefore, approaches that can antagonize or scavenge the released DAMPs, e.g., HMGB1, have been described to ameliorate the inflammation [11,15]. Several pattern recognition receptors and their downstream signaling pathways represent the next targets in mediating inflammation [25]. Toll-like receptors (TLRs) are among the well-established PRRs known to upregulate inflammation [26]. Various pro-inflammatory mediators, such as cytokines (IL-1β, TNF, IL-6, IL-12, IL-17, IL-23, etc.) and chemokines (MCP-1, MIP-1β, and CCL5) acting through their receptors and various adhesion molecules expressed on the surface of endothelial cells and infiltrating leukocytes, are also targets of modulation [27]. Various cellular targets, such as depletion of neutrophils or monocytes/macrophages, also downregulate inflammation [28,29,30]. Moreover, promoting microglial M2 polarization can afford neuroprotection after SAH [31]. Strategies aimed at promoting T-helper cells with anti-inflammatory roles, such Th2, Tregs opposed to Th1, and Th17, could be beneficial in reducing inflammation [32,33,34] and neuroprotective [33,34] via preventing BBB disruption [32,35], preserving endothelial function [35], and shifting microglia/macrophage response toward the M2 phenotype [36]. Adoptive transfer of various peripheral circulating cells with anti-inflammatory potential could also be considered to ameliorate the inflammation. Interestingly, several approaches aimed at modulating the neuro-immune communication, e.g., by vagus nerve stimulation or modulation of α7-nicotinic acid receptor-mediated effects, may be targeted for a reduction in post-aSAH inflammation [37,38]. Besides these, the role of immune checkpoint inhibitors may be evaluated to modulate inflammation after SAH [27]. Modulation of the inflammatory immune milieu by administering stem cell therapies [39] or stem-cell-derived extracellular vesicles [31,39,40] is another emerging frontier in this field that could be used to improve the post-SAH clinical outcome.

## Data Availability

Not applicable.
